# A New Approach to Fuzzy TOPSIS Method Based on Entropy Measure under Spherical Fuzzy Information

**DOI:** 10.3390/e21121231

**Published:** 2019-12-16

**Authors:** Omar Barukab, Saleem Abdullah, Shahzaib Ashraf, Muhammad Arif, Sher Afzal Khan

**Affiliations:** 1 Faculty of Computing and Information Technology, P.O. Box 411, King Abdulaziz University, Rabigh 21911, Jeddah, Saudi Arabia; obarukab@kau.edu.sa; 2 Faculty of Physical and Numerical Sciences, Abdul Wali Khan University, Mardan 23200, Pakistan; shahzaibashraf@awkum.edu.pk (S.A.); marifmaths@awkum.edu.pk (M.A.); sher.afzal@awkum.edu.pk (S.A.K.)

**Keywords:** spherical fuzzy set, spherical fuzzy entropy measure, extended TOPSIS method, group decision making problems

## Abstract

Spherical fuzzy set (SFS) is one of the most important and extensive concept to accommodate more uncertainties than existing fuzzy set structures. In this article, we will describe a novel enhanced TOPSIS-based procedure for tackling multi attribute group decision making (MAGDM) issues under spherical fuzzy setting, in which the weights of both decision-makers (DMs) and criteria are totally unknown. First, we study the notion of SFSs, the score and accuracy functions of SFSs and their basic operating laws. In addition, defined the generalized distance measure for SFSs based on spherical fuzzy entropy measure to compute the unknown weights information. Secondly, the spherical fuzzy information-based decision-making technique for MAGDM is presented. Lastly, an illustrative example is delivered with robot selection to reveal the efficiency of the proposed spherical fuzzy decision support approach, along with the discussion of comparative results, to prove that their results are feasible and credible.

## 1. Introduction

In recent research environment, multi-attribute group decision making (MAGDM) has played a vital role in the decision support systems [1,2,3,4,5,6,7]. Robot selection for the manufacturing units are multi-functional group decision making problems, which are often to resolved by an unprogrammed decision making techniques and involvement of the long period contract with the company. A decision group contains various decision makers/analysis such as development, research, engineering and economic. In fact, the interest of single decision maker may not be same. The final result in group decision making (GDM) method may be essentially changed by the importance level of each decision maker. The growth of multi-functional team involvement in robot selection and estimation particularly affected on buying firm with efficiency. A major issue in decision method is, how to represent the attribute value. The issue in decision making problem arises due to crisp numbers. Because in some cases it is difficult to prove the attribute by using crisp set. So, the decision makers can make choices at a special level. The fuzzy set theory has been implemented in various field such as management, engineering, social sciences to resolve group decision making issues, which involve uncertainty and vagueness in data. The application of fuzzy set theory has remarkable significance in decision making problems.

There are lot of problems arises in decision making due to have uncertainty. To resolve these issues, Zadeh developed the idea of fuzzy set (FS) in 1965 [8]. The concept of fuzzy set is associated with membership grade of the components based on the interval [0,1]. Numerous properties of the Zadeh theory of fuzzy set is noticeable. In the framework of fuzzy set theory, we deal with the problem of making decisions that distinguish the items of a certain universe into more than one appropriate category has been examined. Atanassov’s determined that there are lot of deficiencies in FS. He observed that the concept of negative membership grade might also occurs there, which is vital factor while organizing the entirely recommended pattern and effects of the problems. This type of grade is accurately introduced by intuitionistic fuzzy (IF) set as a substitute of correct values. The components of Atanassov’s IF set [9] is presented in an ordered pair that comprises of characteristic of positive and negative membership grades which follows the condition that sum of both stated functions is less than or equal to one.

There are some situations, where the sum of both positive and negative membership grades are greater than 1, unlike the cases capture in IF sets. Then, the Pythagorean fuzzy (PyF) set are proposed by Yager [10], which are characterized by the positive and negative membership grades satisfies the condition that square sum of the stated functions is less than or equal to one. Many researcher got the attraction and proposed many application of PyF set in decision making. Rahman et al. [11] introduced the geometric aggregation operators for the group decision-making problem with the interval valued PyF set environment. Liang and Xu [12] proposed the notion of hesitant PyF sets and apply TOPSIS method for energy project selection model. Rahman et al. [13] introduced new algorithm for the MAGDM problem using Einstein aggregation operator under PyF information. Utilizing the notion of the immediate probabilities, Garg [14] introduced a series of aggregation operators under PyF information. Garg [15] developed the generalized geometric aggregation operators utilizing Einstein norms for multi-attribute group decision-making problems. Wei and Lu [16] developed the power aggregation operators to deal with a MAGDM problems. Xu et al. [17] launched the induced generalized OWA operators for PyF information. Xue et al. [18] developed the LINMAP technique to track the best investment company in railway projects using PyF information. Yager [19] launched the weighted averaging, geometric, ordered averaging and ordered geometric aggregation operators for PyF information. Perez-Dominguez [20] developed a multi-objective optimization based on the ratio analysis (MOORA) with PyF set information and applied it to decision making problems. Khan et al. [21] proposed the Dombi aggregation operators using Dombi norm for PyF information and discussed their application in decision making. Nguyen and Garg [22], proposed the exponential based similarity measure for PyFS. Garg [23], developed the neutrality geometric operations under PyF information. Athira et al. [24] presented the entropy and distance measure of Pyf soft set and discussed their application in decision making.

Ashraf and Abdullah [25] present the novel idea of spherical fuzzy (SF) set to generalized the concept of PyF set and picture fuzzy (PF) set [26] by considering the neutral membership grade with condition that square sum of the positive, neutral and negative membership grades are less than or equal to one. As a generalization of all the existing structure of FSs, SF set got much more attention of the researchers to deal with uncertainty in decision making problems. Ashraf et al. [27] proposed the algebraic aggregation operators (AOp) using algebraic t-norm and t-conorm to deal with uncertainty in decision making problems (DMP). In [28] Ashraf el al. proposed the series of Dombi AOp for SF information based on the Dombi norms to aggregate the attributes information to sort the best alternative in DMPs. Jin et al. [29] proposed the logarithmic AOp using basic logarithmic operational laws and discussed their application in real world DMP. Ashraf et al. [30] proposed the decision making technique using the concept of distance measure under SF sets. Ashraf et al. [31] proposed the SF set representation of SF t-norm and t-conorm. GRA method for SF information using the concept of linguistic SF set is proposed by Ashraf et al. [32]. Kutlu and Kahraman [33] proposed the decision making technique of WASPAS and in [34] proposed the VIKOR technique utilizing the SF information and also discussed their applications in DMPs. Zeng et al. [35] proposed the SF rough set model and discuss its application in DMP using TOPSIS method. Rafiq et al. [36] proposed the cosine similarity measures for SF information.

Over the years, numerous decision making procedures have been introduced in the literature, of which technique for order preference by similarity to ideal solution (TOPSIS) is one of the extensively and efficient used famous methods. Hwang and Yoon [37] presented the TOPSIS to deal multi-attribute DMPs. Under which the alternative is the smallest distance from the positive ideal solution (PIS) and the furthest distance from the negative ideal solution (NIS) in DMPs is the best alternative. In [38], Chen presented the TOPSIS using FS environment to solve the DMPs. In recent time periods, numerous scholars got attraction and apply TOPSIS to real life DMPs under different extended structures of FS [39,40,41,42,43,44,45,46,47,48,49,50,51,52,53] in the fields of decision sciences [50,54,55]. It is also to be mentioned here that the existing TOPSIS procedures [39,40,41,42,43,44,45,46,47,48,49,50] face the drawback that in solving DMPs, either DMs weights are known [46] or criteria weights are known [43,49] or both [39,41,49,50,54,55]. Some scholars allocated unknown weight information about DMs [56,57] in which criteria weights are known. Instead, some researchers handled unknown criteria weights with known weight data of DMs in MCGDM problems. Though the authors’ concerns, there can be no such tool available in the literary works to address MCGDM problems where the weight data of DMs and criteria are completely unknown within SF information.

Motivated by the above discussion, we plan to introduce a new expanded TOPSIS procedure under spherical fuzzy setting, in order to benefit of the advantages of the TOPSIS method and spherical fuzzy sets. As, the generalized form of the existing structure of fuzzy sets such as IF set, PyF sets, and PF sets is the spherical fuzzy set, thus, SF sets to address more uncertainty compared to FS, IF set, PyF set, and PF set. Therefore, in this paper, a novel improved TOPSIS-based method is established to address with such circumstances of unknown weight information of both DMs and criteria weights and to solve the MAGDM problem after compute all the weights. In order to solve the DMPs, choosing the ideal opinion, which is better connected to each DMs matrix, is quite essential. In the presented procedure, ideal opinion is nominated under SF average method. Generalized distance measure is established to find the differences between two SFSs. In the presented spherical fuzzy TOPSIS (SF-TOPSIS) for solving MAGDM problems, generalized distance measures-based entropy measure is introduced to find out the criteria weights under SF information used in this paper.

The rest of this paper is arranged as following. Section 2 presents some knowledge related to FSs, IFSs, PyFSs, PFSs and SFSs. Section 3 proposed the methodological development of spherical fuzzy entropy measure. In Section 4, established the improved TOPSIS method to address the uncertainty in MAGDM problems. Section 5 reports an illustration example of the designed MAGDM technique for robot selection for manufacturing units and a comparison with existing decision making methods. A conclusion of the paper is drawn in Section 7.

## 2. Preliminaries

In this section, we briefly remember the concepts of fuzzy sets, intuitionistic fuzzy sets, Pythagorean fuzzy sets, picture fuzzy sets and spherical fuzzy sets. These concepts will be used in further study.

**Definition** **1** ([8])**.** *For a fixed set ℑ. A FS ε in ℑ is defined as*

(1) $$\varepsilon =\left\{\langle \varkappa ,\rho_{\varepsilon }\left(\varkappa \right)\rangle |\varkappa \in ℑ\right\},$$

*for each* ϰ∈ℑ, *the positive membership grade* $\rho_{\varepsilon }:ℑ\to \Theta$ *specifies the degree to which the element ϰ belongs to the fuzzy set* ε, *where* $\Theta =\left[0,1\right]$ *be the unit interval.*

**Definition** **2** ([9])**.** *For a fixed set ℑ. An IFS ε in ℑ is defined as*

(2) $$\varepsilon =\left\{\langle \varkappa ,\rho_{\varepsilon }\left(\varkappa \right),{\tilde{n}}_{\varepsilon }\left(\varkappa \right)\rangle |\varkappa \in ℑ\right\},$$

*for each* ϰ∈ℑ, *the positive membership grade* $\rho_{\varepsilon }:ℑ\to \Theta$ *and the negative membership grade* ${\tilde{n}}_{\varepsilon }:ℑ\to \Theta$ *specifies the degree of positive and negative membership of the element ϰ to the Pythagorean fuzzy set* ε, *respectively, where* $\Theta =\left[0,1\right]$ *be the unit interval. Furthermore, it is required that* $0\leq \rho_{\varepsilon }\left(\varkappa \right)+{\tilde{n}}_{\varepsilon }\left(\varkappa \right)\leq 1,$ *for each* ϰ∈ℑ.

**Definition** **3** ([10])**.** *For a fixed set ℑ. A PyFS ε in ℑ is defined as*

(3) $$\varepsilon =\left\{\langle \varkappa ,\rho_{\varepsilon }\left(\varkappa \right),{\tilde{n}}_{\varepsilon }\left(\varkappa \right)\rangle |\varkappa \in ℑ\right\},$$

*for each* ϰ∈ℑ, *the positive membership grade* $\rho_{\varepsilon }:ℑ\to \Theta$ *and the negative membership grade* ${\tilde{n}}_{\varepsilon }:ℑ\to \Theta$ *specifies the degree of positive and negative membership of the element ϰ to the Pythagorean fuzzy set* ε, *respectively, where* $\Theta =\left[0,1\right]$ *be the unit interval. Furthermore, it is required that* $0\leq \rho_{\varepsilon }^{2}\left(\varkappa \right)+{\tilde{n}}_{\varepsilon }^{2}\left(\varkappa \right)\leq 1,$ *for each* ϰ∈ℑ.

**Definition** **4** ([26])**.** *For a fixed set ℑ. A PFS ε in ℑ is defined as*

(4) $$\varepsilon =\left\{\langle \varkappa ,\rho_{\varepsilon }\left(\varkappa \right),{ℸ}_{\varepsilon }\left(\varkappa \right),{\tilde{n}}_{\varepsilon }\left(\varkappa \right)\rangle |\varkappa \in ℑ\right\},$$

*for each* ϰ∈ℑ, *the positive membership* $\rho_{\varepsilon }:ℑ\to \Theta ,$ *neutral membership* ${ℸ}_{\varepsilon }:ℑ\to \Theta$ *and the negative membership* ${\tilde{n}}_{\varepsilon }:ℑ\to \Theta$ *specifies the degree of positive, neutral and negative membership grades of the element ϰ to the picture fuzzy set* ε, *respectively, where* $\Theta =\left[0,1\right]$ *be the unit interval. Furthermore, it is required that* $0\leq \rho_{\varepsilon }\left(\varkappa \right)+{ℸ}_{\varepsilon }\left(\varkappa \right)+{\tilde{n}}_{\varepsilon }\left(\varkappa \right)\leq 1,$ *for each* ϰ∈ℑ.

**Definition** **5** ([25])**.** *For a fixed set ℑ. A SFS ε in ℑ is defined as*

(5) $$\varepsilon =\left\{\langle \varkappa ,\rho_{\varepsilon }\left(\varkappa \right),{ℸ}_{\varepsilon }\left(\varkappa \right),{\tilde{n}}_{\varepsilon }\left(\varkappa \right)\rangle |\varkappa \in ℑ\right\},$$

*for each* ϰ∈ℑ, *the positive membership* $\rho_{\varepsilon }:ℑ\to \Theta ,$ *neutral membership* ${ℸ}_{\varepsilon }:ℑ\to \Theta$ *and the negative membership* ${\tilde{n}}_{\varepsilon }:ℑ\to \Theta$ *specifies the degree of positive, neutral and negative membership grades of the element ϰ to the spherical fuzzy set* ε, *respectively, where* $\Theta =\left[0,1\right]$ *be the unit interval. Furthermore, it is required that* $0\leq \rho_{\varepsilon }^{2}\left(\varkappa \right)+{ℸ}_{\varepsilon }^{2}\left(\varkappa \right)+{\tilde{n}}_{\varepsilon }^{2}\left(\varkappa \right)\leq 1,$ *for each* ϰ∈ℑ.

*Conventionally,* $\upsilon_{\varkappa }=\sqrt{1-\rho_{\varepsilon }^{2}\left(\varkappa \right)-{ℸ}_{\varepsilon }^{2}\left(\varkappa \right)-{\tilde{n}}_{\varepsilon }^{2}\left(\varkappa \right)}$ *is said to be degree of hesitancy of ϰ to* ε. *In what follows, we symbolize by* $\hat{S}ϝ\hat{S}\left(ℑ\right)$ *the collection of all spherical fuzzy sets in ϰ. For simplicity, we shall symbolize the spherical fuzzy number (SFN) by the triplet* $\varepsilon =\left(\rho_{\varepsilon },{ℸ}_{\varepsilon },{\tilde{n}}_{\varepsilon }\right).$

**Remark** **1.** *If we put* ${ℸ}_{\varepsilon }=0$ *in Equation (5). than spherical fuzzy set is reduced to Pythagorean fuzzy set. In other words we say that each Pythagorean fuzzy set is spherical fuzzy set but conversely is not true.*

Also we can say that

**Remark** **2.** *Every picture fuzzy set is the spherical fuzzy set but conversely is not true.*

Hence form above remarks we can say that Pythagorean and picture fuzzy sets are the particular case of spherical fuzzy set.

Let $\varepsilon_{1},\varepsilon_{2}\in \hat{S}ϝ\hat{S}\left(ℑ\right).$ Ashraf and Abdullah [25] defined the following notions:

(1) $\varepsilon_{1}⊑\varepsilon_{2}$ if and only if $\rho_{\varepsilon_{1}}\left(\varkappa \right)\leq \rho_{\varepsilon_{2}}\left(\varkappa \right),{ℸ}_{\varepsilon_{1}}\left(\varkappa \right)\leq {ℸ}_{\varepsilon_{2}}\left(\varkappa \right)$ and ${\tilde{n}}_{\varepsilon_{1}}\left(\varkappa \right)\geq {\tilde{n}}_{\varepsilon_{2}}\left(\varkappa \right)$ for each ϰ∈ℑ. Clearly $\varepsilon_{1}=\varepsilon_{2}$ if $\varepsilon_{1}⊑\varepsilon_{2}$ and $\varepsilon_{2}⊑\varepsilon_{1}.$
(2) $\varepsilon_{1}⊓\varepsilon_{2}=\left\{\min\left(\rho_{\varepsilon_{1}}\left(\varkappa \right),\rho_{\varepsilon_{2}}\left(\varkappa \right)\right),\min\left({ℸ}_{\varepsilon_{1}}\left(\varkappa \right),{ℸ}_{\varepsilon_{2}}\left(\varkappa \right)\right),\max\left({\tilde{n}}_{\varepsilon_{1}}\left(\varkappa \right),{\tilde{n}}_{\varepsilon_{2}}\left(\varkappa \right)\right)|\varkappa \in ℑ\right\},$
(3) $\varepsilon_{1}⊔\varepsilon_{2}=\left\{\max\left(\rho_{\varepsilon_{1}}\left(\varkappa \right),\rho_{\varepsilon_{2}}\left(\varkappa \right)\right),\min\left({ℸ}_{\varepsilon_{1}}\left(\varkappa \right),{ℸ}_{\varepsilon_{2}}\left(\varkappa \right)\right),\min\left({\tilde{n}}_{\varepsilon_{1}}\left(\varkappa \right),{\tilde{n}}_{\varepsilon_{2}}\left(\varkappa \right)\right)|\varkappa \in ℑ\right\},$
(4) $\varepsilon_{1}^{c}=\left\{{\tilde{n}}_{\varepsilon_{1}}\left(\varkappa \right),{ℸ}_{\varepsilon_{1}}\left(\varkappa \right),\rho_{\varepsilon_{1}}\left(\varkappa \right)|\varkappa \in ℑ\right\},$

where $\varepsilon_{1},\varepsilon_{2}\in \hat{S}ϝ\hat{S}\left(ℑ\right)$ and ϰ∈ℑ.

Ashraf and Abdullah proposed the operations for spherical fuzzy numbers. Here we describe three cases to discuss the validation of the proposed operators for dealing the spherical fuzzy informations.

**Definition** **6** ([25])**.** *Let* $\varepsilon_{1}=\left\{\rho_{\varepsilon_{1}}\left(\varkappa \right),{ℸ}_{\varepsilon_{1}}\left(\varkappa \right),{\tilde{n}}_{\varepsilon_{1}}\left(\varkappa \right)\right\}$ *and* $\varepsilon_{2}=\left\{\rho_{\varepsilon_{2}}\left(\varkappa \right),{ℸ}_{\varepsilon_{2}}\left(\varkappa \right),{\tilde{n}}_{\varepsilon_{2}}\left(\varkappa \right)\right\}\in \hat{S}ϝN\left(ℑ\right)$ *with* Φ>0. *Then, the operational rules are as follows:*

*(1)*  $\varepsilon_{1}⊗\varepsilon_{2}=\left\{\rho_{\varepsilon_{1}}\rho_{\varepsilon_{2}},{ℸ}_{\varepsilon_{1}}{ℸ}_{\varepsilon_{2}},\sqrt{{\tilde{n}}_{\varepsilon_{1}}^{2}+{\tilde{n}}_{\varepsilon_{2}}^{2}-{\tilde{n}}_{\varepsilon_{1}}^{2}{\tilde{n}}_{\varepsilon_{2}}^{2}}\right\};$
*(2)*  $\varepsilon_{1}⊕\varepsilon_{2}=\left\{\sqrt{\rho_{\varepsilon_{1}}^{2}+\rho_{\varepsilon_{2}}^{2}-\rho_{\varepsilon_{1}}^{2}\rho_{\varepsilon_{2}}^{2}},{ℸ}_{\varepsilon_{1}}{ℸ}_{\varepsilon_{2}},{\tilde{n}}_{\varepsilon_{1}}{\tilde{n}}_{\varepsilon_{2}}\right\};$
*(3)*  $\varepsilon_{1}^{\Phi }=\left\{{\left(\rho_{\varepsilon_{1}}\right)}^{\Phi },{\left({ℸ}_{\varepsilon_{1}}\right)}^{\Phi },\sqrt{1-{\left(1-{\tilde{n}}_{\varepsilon_{1}}^{2}\right)}^{\Phi }}\right\};$
*(4)*  $\Phi \cdot \varepsilon_{1}=\left\{\sqrt{1-{\left(1-\rho_{\varepsilon_{1}}^{2}\right)}^{\Phi }},{\left({ℸ}_{\varepsilon_{1}}\right)}^{\Phi },{\left({\tilde{n}}_{\varepsilon_{1}}\right)}^{\Phi }\right\}.$

**Definition** **7** ([25])**.** *Let* $\varepsilon_{g}=\left\{\rho_{\varepsilon_{g}}\left(\varkappa \right),{ℸ}_{\varepsilon_{g}}\left(\varkappa \right),{\tilde{n}}_{\varepsilon_{g}}\left(\varkappa \right)\right\}$$\in \hat{S}ϝN\left(ℑ\right)$$\left(g=1,2,3,\ldots ,\tilde{n}\right)$*. Then*

*(a)*  $\overset{ˇ}{s}\overset{ˇ}{c}\left(\varepsilon_{g}\right)=\frac{1}{3}\left(2+\rho_{\varepsilon_{g}}-{ℸ}_{\varepsilon_{g}}-{\tilde{n}}_{\varepsilon_{g}}\right)\in \left[0,1\right]$*is said to be score value of*$\varepsilon_{g}.$
*(b)*  $\tilde{a}\overset{ˇ}{c}\left(\varepsilon_{g}\right)=\left(\rho_{\varepsilon_{g}}^{2}+{ℸ}_{\varepsilon_{g}}^{2}+{\tilde{n}}_{\varepsilon_{g}}^{2}\right)\in \left[0,1\right]$*is said to be accuracy value of*$\varepsilon_{g}.$

**Definition** **8** ([25])**.** *Let* $\varepsilon_{1}=\left\{\rho_{\varepsilon_{1}}\left(\varkappa \right),{ℸ}_{\varepsilon_{1}}\left(\varkappa \right),{\tilde{n}}_{\varepsilon_{1}}\left(\varkappa \right)\right\}$ *and* $\varepsilon_{2}=\left\{\rho_{\varepsilon_{2}}\left(\varkappa \right),{ℸ}_{\varepsilon_{2}}\left(\varkappa \right),{\tilde{n}}_{\varepsilon_{2}}\left(\varkappa \right)\right\}\in \hat{S}ϝN\left(ℑ\right).$ *Then*

*(1)*  *If*$\overset{ˇ}{s}\overset{ˇ}{c}\left(\varepsilon_{1}\right)<\overset{ˇ}{s}\overset{ˇ}{c}\left(\varepsilon_{2}\right)$*then*$\varepsilon_{1}<\varepsilon_{2},$
*(2)*  *If*$\overset{ˇ}{s}\overset{ˇ}{c}\left(\varepsilon_{1}\right)>\overset{ˇ}{s}\overset{ˇ}{c}\left(\varepsilon_{2}\right)$*then*$\varepsilon_{1}>\varepsilon_{2},$
*(3)*  *If*$\overset{ˇ}{s}\overset{ˇ}{c}\left(\varepsilon_{1}\right)=\overset{ˇ}{s}\overset{ˇ}{c}\left(\varepsilon_{2}\right)$*then*
  *(a)*  $\tilde{a}\overset{ˇ}{c}\left(\varepsilon_{1}\right)<\tilde{a}\overset{ˇ}{c}\left(\varepsilon_{2}\right)$*then*$\varepsilon_{1}<\varepsilon_{2},$
  *(b)*  $\tilde{a}\overset{ˇ}{c}\left(\varepsilon_{1}\right)>\tilde{a}\overset{ˇ}{c}\left(\varepsilon_{2}\right)$*then*$\varepsilon_{1}>\varepsilon_{2},$
  *(c)*  $\tilde{a}\overset{ˇ}{c}\left(\varepsilon_{1}\right)=\tilde{a}\overset{ˇ}{c}\left(\varepsilon_{2}\right)$*then*$\varepsilon_{1}=\varepsilon_{2}.$

**Definition** **9** ([25])**.** *Let* $\varepsilon_{g}=\left\{\rho_{\varepsilon_{g}}\left(\varkappa \right),{ℸ}_{\varepsilon_{g}}\left(\varkappa \right),{\tilde{n}}_{\varepsilon_{g}}\left(\varkappa \right)\right\}$$\in \hat{S}ϝN\left(ℑ\right)$$\left(g=1,2,3,\ldots ,n\right)$*. Then, the Algebraic averaging aggregation operator for* $\hat{S}ϝN\left(ℑ\right)$ *is denoted by* SFWA *and defined as follows:*

(6) $$\begin{array}{ccc} SFEWA\left(\varepsilon_{1},\varepsilon_{2},\varepsilon_{3},\ldots ,\varepsilon_{\tilde{n}}\right) & = & \sum_{g=1}^{n}\kappa_{g}\varepsilon_{g},\\ & = & \left\{\sqrt{1-\Pi_{g=1}^{n}{\left(1-\rho_{\varepsilon_{g}}^{2}\right)}^{\kappa_{g}}},\Pi_{g=1}^{n}{\left({ℸ}_{\varepsilon_{g}}\right)}^{\kappa_{g}},\Pi_{g=1}^{n}{\left({\tilde{n}}_{\varepsilon_{g}}\right)}^{\kappa_{g}}\right\} \end{array}$$

*where* $\kappa_{g}\left(g=1,2,\ldots ,n\right)$ *represents the weights of* $\varepsilon_{g}\left(g=1,2,3,\ldots ,n\right)$ *with* $\kappa_{g}\geq 0$ *and* $\sum_{g=1}^{n}\kappa_{g}=1.$

## 3. Methodological Development of Spherical Fuzzy Entropy Measure

This section proposed the generalized distance and weighted generalized distance measures for spherical fuzzy sets. After that, utilizing the generalized distance measures, we proposed the novel entropy measure for SFS to measure the fuzziness of SFS.

### 3.1. SF Distance Measure

**Definition** **10.** *Let for any* $ℶ=\left\{{ℶ}_{1},{ℶ}_{2},\ldots ,{ℶ}_{n}\right\},∁=\left\{{∁}_{1},{∁}_{2},\ldots ,{∁}_{n}\right\}\in \hat{S}ϝS\left(ℑ\right),$ *where* ${ℶ}_{g}=\left\{\rho_{{ℶ}_{g}}\left(\varkappa \right),{ℸ}_{{ℶ}_{g}}\left(\varkappa \right),{\tilde{n}}_{{ℶ}_{g}}\left(\varkappa \right)\right\}$ *and* ${∁}_{g}=\left\{\rho_{{∁}_{g}}\left(\varkappa \right),{ℸ}_{{∁}_{g}}\left(\varkappa \right),{\tilde{n}}_{{∁}_{g}}\left(\varkappa \right)\right\}$ $g=\left\{1,2,3,\ldots ,n\right\}.$ *Then, the generalized distance measure (GDM) between ℶ and ∁ is defined for any* $\Phi >0\left(\in R\right)$ *as*

(7) $$d_{G}\left(ℶ,∁\right)={\left(\frac{1}{2n}\sum_{g=1}^{n}\left(\begin{array}{c} {\left|{\left(\rho_{{ℶ}_{g}}\right)}^{2}-{\left(\rho_{{∁}_{g}}\right)}^{2}\right|}^{\Phi }+{\left|{\left({ℸ}_{{ℶ}_{g}}\right)}^{2}-{\left({ℸ}_{{∁}_{g}}\right)}^{2}\right|}^{\Phi }+\\ {\left|{\left({\tilde{n}}_{{ℶ}_{g}}\right)}^{2}-{\left({\tilde{n}}_{{∁}_{g}}\right)}^{2}\right|}^{\Phi } \end{array}\right)\right)}^{\frac{1}{\Phi }}.$$

**Definition** **11.** *Let for any* $ℶ=\left\{{ℶ}_{1},{ℶ}_{2},\ldots ,{ℶ}_{n}\right\},∁=\left\{{∁}_{1},{∁}_{2},\ldots ,{∁}_{n}\right\}\in \hat{S}ϝS\left(ℑ\right),$ *where* ${ℶ}_{g}=\left\{\rho_{{ℶ}_{g}}\left(\varkappa \right),{ℸ}_{{ℶ}_{g}}\left(\varkappa \right),{\tilde{n}}_{{ℶ}_{g}}\left(\varkappa \right)\right\}$ *and* ${∁}_{g}=\left\{\rho_{{∁}_{g}}\left(\varkappa \right),{ℸ}_{{∁}_{g}}\left(\varkappa \right),{\tilde{n}}_{{∁}_{g}}\left(\varkappa \right)\right\}$ $g=\left\{1,2,3,\ldots ,n\right\}.$ *Then, the weighted generalized distance measure (WGDM) between ℶ and ∁ is defined for any* $\Phi >0\left(\in R\right)$ *as*

(8) $$d_{WG}\left(ℶ,∁\right)={\left(\frac{1}{2n}\sum_{g=1}^{n}\kappa_{g}\left(\begin{array}{c} {\left|{\left(\rho_{{ℶ}_{g}}\right)}^{2}-{\left(\rho_{{∁}_{g}}\right)}^{2}\right|}^{\Phi }+{\left|{\left({ℸ}_{{ℶ}_{g}}\right)}^{2}-{\left({ℸ}_{{∁}_{g}}\right)}^{2}\right|}^{\Phi }+\\ {\left|{\left({\tilde{n}}_{{ℶ}_{g}}\right)}^{2}-{\left({\tilde{n}}_{{∁}_{g}}\right)}^{2}\right|}^{\Phi } \end{array}\right)\right)}^{\frac{1}{\Phi }},$$

*where* $\kappa_{g}\left(g=1,2,\ldots ,n\right)$ *represents the weights with condition that* $\kappa_{g}\geq 0$ *and* $\sum_{g=1}^{n}\kappa_{g}=1.$

**Remark** **3.**

*(1)*  *If* Φ=1, *then, the distance defined in Definitions 10 and 11 reduced to Hamming distance.*
*(2)*  *If* Φ=2, *then, the distance defined in Definitions 10 and 11 reduced to Euclidean distance.*
*(3)*  *If* Φ=+∞, *then, the distance defined in Definitions 10 and 11 reduced to Chebychev distance.*

**Definition** **12.** *Let* $\varepsilon_{g}=\left\{\rho_{\varepsilon_{g}}\left(\varkappa \right),{ℸ}_{\varepsilon_{g}}\left(\varkappa \right),{\tilde{n}}_{\varepsilon_{g}}\left(\varkappa \right)\right\}\in \hat{S}ϝN\left(ℑ\right)$ $g=\left\{1,2,\right\}$ *. Then the GDM defined in Definition 10 reduced as follows*

(9) $$d_{G}\left(\varepsilon_{1},\varepsilon_{2}\right)={\left(\frac{1}{2}\left(\begin{array}{c} {\left|{\left(\rho_{\varepsilon_{1}}\right)}^{2}-{\left(\rho_{\varepsilon_{2}}\right)}^{2}\right|}^{\Phi }+{\left|{\left({ℸ}_{\varepsilon_{1}}\right)}^{2}-{\left({ℸ}_{\varepsilon_{2}}\right)}^{2}\right|}^{\Phi }+\\ {\left|{\left({\tilde{n}}_{\varepsilon_{1}}\right)}^{2}-{\left({\tilde{n}}_{\varepsilon_{2}}\right)}^{2}\right|}^{\Phi } \end{array}\right)\right)}^{\frac{1}{\Phi }},\;\Phi >0\left(\in R\right).$$

For any two $\varepsilon_{1},\varepsilon_{2}\in \hat{S}ϝN\left(ℑ\right),$ the above defined GDMs satisfied the following properties

(1) $0\leq d\left(\varepsilon_{1},\varepsilon_{2}\right)\leq 1,$
(2) $d\left(\varepsilon_{1},\varepsilon_{2}\right)=1,$ iff $\varepsilon_{1}=\varepsilon_{2},$
(3) $d\left(\varepsilon_{1},\varepsilon_{2}\right)=d\left(\varepsilon_{2},\varepsilon_{1}\right).$

### 3.2. SF Entropy Measure

In this section, we propose a new entropy measure for SFS based distance measure, for this we follows the concept of Guo and Song [58].

**Definition** **13.** *Let for any* $ℶ=\left\{{ℶ}_{1},{ℶ}_{2},\ldots ,{ℶ}_{n}\right\}\in \hat{S}ϝS\left(\varkappa \right),$ *where* ${ℶ}_{g}=\left\{\rho_{{ℶ}_{g}}\left(\varkappa \right),{ℸ}_{{ℶ}_{g}}\left(\varkappa \right),{\tilde{n}}_{{ℶ}_{g}}\left(\varkappa \right)\right\}$ *is SFNs for each* $g=\left\{1,2,3,\ldots ,n\right\}.$ *Then, the entropy measure for SFS ℶ is defined as*

(10) $$E\left(ℶ\right)=\frac{1}{n}\sum_{g=1}^{n}\left[\left\{1-d\left({ℶ}_{g},{ℶ}_{g}^{c}\right)\right\}\frac{1+{\left(\upsilon_{{ℶ}_{g}}\right)}^{2}}{2}\right].$$

**Theorem** **1.** *Let for any* $ℶ=\left\{{ℶ}_{1},{ℶ}_{2},\ldots ,{ℶ}_{n}\right\},∁=\left\{{∁}_{1},{∁}_{2},\ldots ,{∁}_{n}\right\}\in \hat{S}ϝS\left(ℑ\right),$ *where* ${ℶ}_{g}=\left\{\rho_{{ℶ}_{g}}\left(\varkappa \right),{ℸ}_{{ℶ}_{g}}\left(\varkappa \right),{\tilde{n}}_{{ℶ}_{g}}\left(\varkappa \right)\right\}$ *and* ${∁}_{g}=\left\{\rho_{{∁}_{g}}\left(\varkappa \right),{ℸ}_{{∁}_{g}}\left(\varkappa \right),{\tilde{n}}_{{∁}_{g}}\left(\varkappa \right)\right\}$ *are SFNs for each* $g=\left\{1,2,3,\ldots ,n\right\}.$ *Then the entropy measures* $E\left(ℶ\right)$ *and* $E\left(∁\right)$ *satisfies the following properties:*

*(1)*  $E\left(ℶ\right)=0$ *iff ℶ is the crisp set,*
*(2)*  $E\left(ℶ\right)\leq$ $E\left(∁\right)$ *if* ℶ≤∁, *that is* $\rho_{ℶ}\left(\varkappa \right)\leq \rho_{∁}\left(\varkappa \right),{ℸ}_{ℶ}\left(\varkappa \right)\leq {ℸ}_{∁}\left(\varkappa \right)$ *and* ${\tilde{n}}_{ℶ}\left(\varkappa \right)\geq {\tilde{n}}_{∁}\left(\varkappa \right)$ *for each* ϰ∈ℑ,
*(3)*  $E\left(ℶ\right)\leq$ $E\left({ℶ}^{c}\right).$

**Proof.** 

(1) For a crisp set, we have $\rho_{ℶ}\left(\varkappa \right)=1,$${ℸ}_{ℶ}\left(\varkappa \right)=0$ and ${\tilde{n}}_{ℶ}\left(\varkappa \right)=0$ for each ϰ∈ℑ. Hence, $E\left(ℶ\right)=0.$
  Conversely, suppose that $E\left(ℶ\right)=0.$
  Since $\rho_{ℶ}\left(\varkappa \right),$${ℸ}_{ℶ}\left(\varkappa \right)$ and ${\tilde{n}}_{ℶ}\left(\varkappa \right)\in \left[0,1\right]$ for each ϰ∈ℑ,$1+\upsilon_{ℶ}^{2}\neq 0.$ Therefore,
  (11) $$1-d\left(ℶ,{ℶ}^{c}\right)=0$$
  Only possibility if $\rho_{ℶ}\left(\varkappa \right)\leq 1$ and ${\tilde{n}}_{ℶ}\left(\varkappa \right)\leq 1.$ Equation 11 holds when $\rho_{ℶ}\left(\varkappa \right)=1$ or ${\tilde{n}}_{ℶ}\left(\varkappa \right)=1$ for each ϰ∈ℑ.
  Hence, *ℶ* is the crisp set.
(2) Suppose ℶ≤∁. Then, $\rho_{ℶ}\left(\varkappa \right)\leq \rho_{∁}\left(\varkappa \right),{ℸ}_{ℶ}\left(\varkappa \right)\leq {ℸ}_{∁}\left(\varkappa \right)$ and ${\tilde{n}}_{ℶ}\left(\varkappa \right)\geq {\tilde{n}}_{∁}\left(\varkappa \right)$ for each ϰ∈ℑ.
  For this we have to show that $E\left(∁\right)-E\left(ℶ\right)\geq 0.$
  $$\begin{array}{ccc} E\left(∁\right)-E\left(ℶ\right) & = & \frac{1}{2n}\sum_{g=1}^{n}\left[\left(1-\left|\rho_{∁}^{2}-{\tilde{n}}_{∁}^{2}\right|\right)\left(2-\rho_{∁}^{2}-{ℸ}_{∁}^{2}-{\tilde{n}}_{∁}^{2}\right)-\left(1-\left|\rho_{ℶ}^{2}-{\tilde{n}}_{ℶ}^{2}\right|\right)\left(2-\rho_{ℶ}^{2}-{ℸ}_{ℶ}^{2}-{\tilde{n}}_{ℶ}^{2}\right)\right]\\ \; & = & \frac{1}{2n}\sum_{g=1}^{n}\left[\left(1+\left(\rho_{∁}^{2}-{\tilde{n}}_{∁}^{2}\right)\right)\left(2-\rho_{∁}^{2}-{ℸ}_{∁}^{2}-{\tilde{n}}_{∁}^{2}\right)-\left(1+\left(\rho_{ℶ}^{2}-{\tilde{n}}_{ℶ}^{2}\right)\right)\left(2-\rho_{ℶ}^{2}-{ℸ}_{ℶ}^{2}-{\tilde{n}}_{ℶ}^{2}\right)\right]\\ \; & = & \frac{1}{2n}\sum_{g=1}^{n}\left[\begin{array}{c} \left(2+\rho_{∁}^{2}-{ℸ}_{∁}^{2}-3{\tilde{n}}_{∁}^{2}-\rho_{∁}^{2}{ℸ}_{∁}^{2}+{ℸ}_{∁}^{2}{\tilde{n}}_{∁}^{2}-\rho_{∁}^{4}+{\tilde{n}}_{∁}^{4}\right)-\\ \left(2+\rho_{ℶ}^{2}-{ℸ}_{ℶ}^{2}-3{\tilde{n}}_{ℶ}^{2}-\rho_{ℶ}^{2}{ℸ}_{ℶ}^{2}+{ℸ}_{ℶ}^{2}{\tilde{n}}_{ℶ}^{2}-\rho_{ℶ}^{4}+{\tilde{n}}_{ℶ}^{4}\right) \end{array}\right]\\ \; & = & \frac{1}{2n}\sum_{g=1}^{n}\left[\begin{array}{c} \left(\rho_{∁}^{2}-\rho_{ℶ}^{2}\right)+\left({ℸ}_{ℶ}^{2}-{ℸ}_{∁}^{2}\right)+3\left({\tilde{n}}_{ℶ}^{2}-{\tilde{n}}_{∁}^{2}\right)+\left(\rho_{ℶ}^{2}{ℸ}_{ℶ}^{2}-\rho_{∁}^{2}{ℸ}_{∁}^{2}\right)+\\ \left({ℸ}_{∁}^{2}{\tilde{n}}_{∁}^{2}-{ℸ}_{ℶ}^{2}{\tilde{n}}_{ℶ}^{2}\right)+\left(\rho_{ℶ}^{4}-\rho_{∁}^{4}\right)+\left({\tilde{n}}_{∁}^{4}-{\tilde{n}}_{ℶ}^{4}\right) \end{array}\right]\geq 0 \end{array}$$
  Since, all power are even, then implies that
  $$E\left(ℶ\right)\leq E\left(∁\right).$$
(3) Since, we have
  $$\begin{array}{ccc} E\left(ℶ\right) & = & \frac{1}{n}\sum_{g=1}^{n}\left[\left\{1-d\left({ℶ}_{g},{ℶ}_{g}^{c}\right)\right\}\frac{1+{\left(\upsilon_{{ℶ}_{g}}\right)}^{2}}{2}\right]\\ & = & \frac{1}{n}\sum_{g=1}^{n}\left[\left\{1-\left|\rho_{ℶ}^{2}-{\tilde{n}}_{ℶ}^{2}\right|\right\}\frac{2-\rho_{ℶ}^{2}-{ℸ}_{ℶ}^{2}-{\tilde{n}}_{ℶ}^{2}}{2}\right]\\ & = & \frac{1}{n}\sum_{g=1}^{n}\left[\left\{1-\left|{\tilde{n}}_{ℶ}^{2}-\rho_{ℶ}^{2}\right|\right\}\frac{2-{\tilde{n}}_{ℶ}^{2}-{ℸ}_{ℶ}^{2}-\rho_{ℶ}^{2}}{2}\right]\\ & = & E\left({ℶ}^{c}\right). \end{array}$$

 □

## 4. SF Improved TOPSIS

### 4.1. Spherical Fuzzy MAGDM Problem

We propose a technique to solve the MAGDM problems in term of spherical fuzzy informations. The MAGDM problems can be addressed in the form of decision matrix where the columns represent the set of attributes and the rows symbolize alternatives. Thus, for decision matrix $D_{n\times m}$, consider a set of *n* alternatives $\left\{S_{1},S_{2},S_{3},\ldots ,S_{n}\right\}$ and *m* criteria/attributes $\left\{f_{1},f_{2},f_{3},\ldots ,f_{m}\right\}$. The unknown weight vector of *m* criteria/attributes is denoted by $W=\left\{\rho_{1},\rho_{2},\rho_{3},\ldots ,\rho_{m}\right\}$ with subject to $\rho_{g}\in \left[0,1\right]$ such that $\sum_{g=1}^{m}\rho_{g}=1.$ Supposed the spherical fuzzy decision matrix is denoted by $D^{\left(k\right)}={\left[\varepsilon_{ij}^{\left(k\right)}\right]}_{\tilde{n}\times m}={\langle \rho_{\varepsilon_{ij}}^{\left(k\right)},{ℸ}_{\varepsilon_{ij}}^{\left(k\right)},{\tilde{n}}_{\varepsilon_{ij}}^{\left(k\right)}\rangle }_{\tilde{n}\times m},$k∈1,2,…,e, where $\rho_{ij}$ represents the degree of the alternative gratifies the criteria $f_{j}$ considered by decision makers (DMs), ${ℸ}_{ij}$ represents the degree of the alternative is neutral for the criteria $f_{j}$ considered by DMs and ${\tilde{n}}_{ij}$ represents the degree of the alternative doesn’t gratify the criteria $f_{j}$ considered by DMs.

$$D_{n\times m}^{\left(k\right)}=\begin{array}{c} S_{1}\\ S_{2}\\ \vdots \\ S_{n} \end{array}\left(\begin{array}{cccc} f_{1} & f_{2} & \; & f_{m}\\ \langle \rho_{\varepsilon_{11}}^{\left(k\right)},{ℸ}_{\varepsilon_{11}}^{\left(k\right)},{\tilde{n}}_{\varepsilon_{11}}^{\left(k\right)}\rangle & \langle \rho_{\varepsilon_{12}}^{\left(k\right)},{ℸ}_{\varepsilon_{12}}^{\left(k\right)},{\tilde{n}}_{\varepsilon_{12}}^{\left(k\right)}\rangle & \ldots & \langle \rho_{\varepsilon_{1m}}^{\left(k\right)},{ℸ}_{\varepsilon_{1m}}^{\left(k\right)},{\tilde{n}}_{\varepsilon_{1m}}^{\left(k\right)}\rangle \\ \langle \rho_{\varepsilon_{21}}^{\left(k\right)},{ℸ}_{\varepsilon_{21}}^{\left(k\right)},{\tilde{n}}_{\varepsilon_{21}}^{\left(k\right)}\rangle & \langle \rho_{\varepsilon_{22}}^{\left(k\right)},{ℸ}_{\varepsilon_{22}}^{\left(k\right)},{\tilde{n}}_{\varepsilon_{22}}^{\left(k\right)}\rangle & \ldots & \langle \rho_{\varepsilon_{2m}}^{\left(k\right)},{ℸ}_{\varepsilon_{2m}}^{\left(k\right)},{\tilde{n}}_{\varepsilon_{2m}}^{\left(k\right)}\rangle \\ \vdots & \vdots & \ddots & \vdots \\ \langle \rho_{\varepsilon_{n1}}^{\left(k\right)},{ℸ}_{\varepsilon_{n1}}^{\left(k\right)},{\tilde{n}}_{\varepsilon_{n1}}^{\left(k\right)}\rangle & \langle \rho_{\varepsilon_{n2}}^{\left(k\right)},{ℸ}_{\varepsilon_{n2}}^{\left(k\right)},{\tilde{n}}_{\varepsilon_{n2}}^{\left(k\right)}\rangle & \ldots & \langle \rho_{\varepsilon_{nm}}^{\left(k\right)},{ℸ}_{\varepsilon_{nm}}^{\left(k\right)},{\tilde{n}}_{\varepsilon_{nm}}^{\left(k\right)}\rangle \end{array}\right)$$

It should be noted here that in the context of decision-making, all the data about the weights of DMs and criteria are unknown.

### 4.2. SF-TOPSIS Method

The procedure contains three main parts. In the first part, we compute the weights of the decision maker. The second part is discussed, how to compute the weights of the criteria using the proposed entropy measure. The last part is a ranking method based on degree of similarity to ideal solution with PIS and NIS.

To solve the spherical fuzzy MAGDM problem using TOPSIS-based procedure, the following steps are introduced:

**Step-1**  Normalize the decision matrix $D_{n\times m}^{\left(k\right)}$. There are usually two types of attributes/criteria in a MAGDM problem, one is the benefit type criteria and other one is the cost type criteria. To unify the criteria, the cost type criteria convert to benefit type criteria by using the following equation:
  (12) $$N_{ij}^{\left(k\right)}=\left\{\begin{array}{ccc} \left(\rho_{\varepsilon_{ij}},{ℸ}_{\varepsilon_{ij}},{\tilde{n}}_{\varepsilon_{ij}}\right) & if & C_{I}\\ \left({\tilde{n}}_{\varepsilon_{ij}},{ℸ}_{\varepsilon_{ij}},\rho_{\varepsilon_{ij}}\right) & if & C_{II} \end{array}\right.$$
  where $C_{I}$ stands for benefit criterion and $C_{II}$ stands for cost criterion.
**Step-2(a)**  The group decision ideal solution (GDIS) is closer to all the opinion of each single DM’s and therefore, the best GDIS should be computed by taking the averaging of all the opinion of each single DM’s. So, in this step, we compute the GDIS by taking spherical fuzzy weighted average of alternatives value corresponding to the criteria given by the DM’s by considering the same weightage of DM’s values as follows:
  $$GDIS=\left(\begin{array}{cccc} IS_{11} & IS_{12} & \ldots & IS_{1n}\\ IS_{21} & IS_{22} & \ldots & IS_{21}\\ \vdots & \vdots & \ddots & \vdots \\ IS_{m1} & IS_{m1} & \ldots & IS_{mn} \end{array}\right)$$
  where
  $$\begin{array}{ccc} IS_{ij} & = & \sum_{k=1}^{e}\frac{1}{e}N_{ij}^{\left(k\right)}\\ & = & \left\{\sqrt{1-\Pi_{k=1}^{e}{\left(1-{\left(\rho_{\varepsilon_{ij}}^{\left(k\right)}\right)}^{2}\right)}^{\frac{1}{e}}},\Pi_{k=1}^{e}{\left({ℸ}_{\varepsilon_{ij}}^{\left(k\right)}\right)}^{\frac{1}{e}},\Pi_{k=1}^{e}{\left({\tilde{n}}_{\varepsilon_{ij}}^{\left(k\right)}\right)}^{\frac{1}{e}}\right\} \end{array}$$
**Step-2(b)**  Computed the group right ideal decision (GRID) and group left ideal decision (GLID) as follows:
  $$GRID=\left(\begin{array}{cccc} RID_{11} & RID_{12} & \ldots & RID_{1n}\\ RID_{21} & RID_{22} & \ldots & RID_{21}\\ \vdots & \vdots & \ddots & \vdots \\ RID_{m1} & RID_{m1} & \ldots & RID_{mn} \end{array}\right)$$
  where
  $$RID_{ij}=\left\{\left(N_{ij}^{\left(k\right)}\right):\begin{array}{c} \max\\ k \end{array}\left[\overset{ˇ}{s}\overset{ˇ}{c}\left(N_{ij}^{\left(k\right)}\right)\right]\right\},$$
  and
  $$GLID=\left(\begin{array}{cccc} LID_{11} & LID_{12} & \ldots & LID_{1n}\\ LID_{21} & LID_{22} & \ldots & LID_{21}\\ \vdots & \vdots & \ddots & \vdots \\ LID_{m1} & LID_{m1} & \ldots & LID_{mn} \end{array}\right)$$
  where
  $$LID_{ij}=\left\{\left(N_{ij}^{\left(k\right)}\right):\begin{array}{c} \min\\ k \end{array}\left[\overset{ˇ}{s}\overset{ˇ}{c}\left(N_{ij}^{\left(k\right)}\right)\right]\right\}.$$
**Step-2(c)**  In this step, we use the Definition 10 to compute the distance of decision matrix $N_{ij}^{\left(k\right)}$ to GDIS,GRID and GLID. The distances are shown symbolically as: DGDIS,DGRID and DGLID respectively. Where
  $$DGDIS_{i}^{\left(k\right)}={\left(\frac{1}{2n}\sum_{j=1}^{n}\left(\begin{array}{c} {\left|{\left(\rho_{N_{ij}^{\left(k\right)}}\right)}^{2}-{\left(\rho_{IS_{ij}}\right)}^{2}\right|}^{\Phi }+{\left|{\left({ℸ}_{N_{ij}^{\left(k\right)}}\right)}^{2}-{\left({ℸ}_{IS_{ij}}\right)}^{2}\right|}^{\Phi }+\\ {\left|{\left({\tilde{n}}_{N_{ij}^{\left(k\right)}}\right)}^{2}-{\left({\tilde{n}}_{IS_{ij}}\right)}^{2}\right|}^{\Phi } \end{array}\right)\right)}^{\frac{1}{\Phi }},$$
  $$DGRID_{i}^{\left(k\right)}={\left(\frac{1}{2n}\sum_{j=1}^{n}\left(\begin{array}{c} {\left|{\left(\rho_{N_{ij}^{\left(k\right)}}\right)}^{2}-{\left(\rho_{RID_{ij}}\right)}^{2}\right|}^{\Phi }+{\left|{\left({ℸ}_{N_{ij}^{\left(k\right)}}\right)}^{2}-{\left({ℸ}_{RID_{ij}}\right)}^{2}\right|}^{\Phi }+\\ {\left|{\left({\tilde{n}}_{N_{ij}^{\left(k\right)}}\right)}^{2}-{\left({\tilde{n}}_{RID_{ij}}\right)}^{2}\right|}^{\Phi } \end{array}\right)\right)}^{\frac{1}{\Phi }},$$
  $$DGLID_{i}^{\left(k\right)}={\left(\frac{1}{2n}\sum_{j=1}^{n}\left(\begin{array}{c} {\left|{\left(\rho_{N_{ij}^{\left(k\right)}}\right)}^{2}-{\left(\rho_{LID_{ij}}\right)}^{2}\right|}^{\Phi }+{\left|{\left({ℸ}_{N_{ij}^{\left(k\right)}}\right)}^{2}-{\left({ℸ}_{LID_{ij}}\right)}^{2}\right|}^{\Phi }+\\ {\left|{\left({\tilde{n}}_{N_{ij}^{\left(k\right)}}\right)}^{2}-{\left({\tilde{n}}_{LID_{ij}}\right)}^{2}\right|}^{\Phi } \end{array}\right)\right)}^{\frac{1}{\Phi }},$$
  for i=1,2,…,m and k=1,2,…,e.
**Step-2(d)**  In this step, we calculate the closeness indices (CIs) followed the model proposed by Yue [56] as follows:
  $$CI^{\left(k\right)}=\frac{\sum_{i=1}^{m}DGRID_{i}^{\left(k\right)}+\sum_{i=1}^{m}DGLID_{i}^{\left(k\right)}}{\sum_{i=1}^{m}DGDIS_{i}^{\left(k\right)}+\sum_{i=1}^{m}DGRID_{i}^{\left(k\right)}+\sum_{i=1}^{m}DGLID_{i}^{\left(k\right)}}.$$
  For k=1,2,…,e.
**Step-2(e)**  In this step, DMs weights are calculate as follows:
  $$Y^{\left(k\right)}=\frac{CI^{\left(k\right)}}{\sum_{k=1}^{e}CI^{\left(k\right)}}$$
**Step-3(a)**  Computed the weights of attribute by using proposed SF entropy measure, for this calculate the revised group decision (RGDIS) as follows:
  $$\begin{array}{ccc} RIS_{ij} & = & \sum_{k=1}^{e}Y^{\left(k\right)}N_{ij}^{\left(k\right)}\\ & = & \left\{\sqrt{1-\Pi_{k=1}^{e}{\left(1-{\left(\rho_{\varepsilon_{ij}}^{\left(k\right)}\right)}^{2}\right)}^{Y^{\left(k\right)}}},\Pi_{k=1}^{e}{\left({ℸ}_{\varepsilon_{ij}}^{\left(k\right)}\right)}^{Y^{\left(k\right)}},\Pi_{k=1}^{e}{\left({\tilde{n}}_{\varepsilon_{ij}}^{\left(k\right)}\right)}^{Y^{\left(k\right)}}\right\}. \end{array}$$
**Step-3(b)**  Using Equation (10), SF entropy measure corresponding to each attribute is computed as follows:
  $$EA_{j}=E\left(RIS_{1j},RIS_{2j},\ldots ,RIS_{mj}\right),\;j=1,2,\ldots ,n.$$
**Step-3(c)**  Attribute weights are calculate as follows:
  $$\Phi A_{j}=\frac{1-EA_{j}}{n-\Pi_{j=1}^{n}EA_{j}},\;j=1,2,\ldots ,n.$$
**Step-4(a)**  Utilizing the attributes weight vector, the weighted normalized decision matrices are computed as follows:
  $$\begin{array}{ccc} DM{\left(N\right)}_{ij}^{\left(k\right)} & = & \sum_{k=1}^{e}\Phi A_{j}N_{ij}^{\left(k\right)}\\ & = & \left\{\sqrt{1-{\left(1-{\left(\rho_{\varepsilon_{ij}}^{\left(k\right)}\right)}^{2}\right)}^{\Phi A_{j}}},{\left({ℸ}_{\varepsilon_{ij}}^{\left(k\right)}\right)}^{\Phi A_{j}},{\left({\tilde{n}}_{\varepsilon_{ij}}^{\left(k\right)}\right)}^{\Phi A_{j}}\right\}, \end{array}$$
  for each k=1,2,…,e.
**Step-4(b)**  Utilizing the weighted normalized decision matrices $DM{\left(N\right)}_{ij}^{\left(k\right)},$ computed the $PIS^{\left(k\right)}$ and $NIS^{\left(k\right)}$ for each DMs as follows:
  $$PIS^{\left(k\right)}=\left\{\left(DM{\left(N\right)}_{ij}^{\left(k\right)}\right):\begin{array}{c} \max\\ i \end{array}\left[\overset{ˇ}{s}\overset{ˇ}{c}\left(DM{\left(N\right)}_{ij}^{\left(k\right)}\right)\right]\right\},\;j=1,2,\ldots ,n$$
  and
  $$NIS^{\left(k\right)}=\left\{\left(DM{\left(N\right)}_{ij}^{\left(k\right)}\right):\begin{array}{c} \min\\ i \end{array}\left[\overset{ˇ}{s}\overset{ˇ}{c}\left(DM{\left(N\right)}_{ij}^{\left(k\right)}\right)\right]\right\},\;j=1,2,\ldots ,n$$
**Step-4(c)**  Computed the WGDM by using the Definition 11 from $DM{\left(N\right)}^{\left(k\right)}$ to $PIS^{\left(k\right)}$ and $NIS^{\left(k\right)}$ are denoted and defined as follows:
  $$DIS_{i}^{+\left(k\right)}={\left(\frac{1}{2n}\sum_{j=1}^{n}\Phi A_{j}\left(\begin{array}{c} {\left|{\left(\rho_{DM{\left(N\right)}^{\left(k\right)}}\right)}^{2}-{\left(\rho_{PIS^{\left(k\right)}}\right)}^{2}\right|}^{\Phi }+{\left|{\left({ℸ}_{DM{\left(N\right)}^{\left(k\right)}}\right)}^{2}-{\left({ℸ}_{PIS^{\left(k\right)}}\right)}^{2}\right|}^{\Phi }+\\ {\left|{\left({\tilde{n}}_{DM{\left(N\right)}^{\left(k\right)}}\right)}^{2}-{\left({\tilde{n}}_{PIS^{\left(k\right)}}\right)}^{2}\right|}^{\Phi } \end{array}\right)\right)}^{\frac{1}{\Phi }},$$
  and
  $$DIS_{i}^{-\left(k\right)}={\left(\frac{1}{2n}\sum_{j=1}^{n}\Phi A_{j}\left(\begin{array}{c} {\left|{\left(\rho_{DM{\left(N\right)}^{\left(k\right)}}\right)}^{2}-{\left(\rho_{NIS^{\left(k\right)}}\right)}^{2}\right|}^{\Phi }+{\left|{\left({ℸ}_{DM{\left(N\right)}^{\left(k\right)}}\right)}^{2}-{\left({ℸ}_{NIS^{\left(k\right)}}\right)}^{2}\right|}^{\Phi }+\\ {\left|{\left({\tilde{n}}_{DM{\left(N\right)}^{\left(k\right)}}\right)}^{2}-{\left({\tilde{n}}_{NIS^{\left(k\right)}}\right)}^{2}\right|}^{\Phi } \end{array}\right)\right)}^{\frac{1}{\Phi }}$$
  for each i=1,2,…,m.
**Step-4(d)**  Revised closeness indices (RCIs) for each DM’s are computed as follows:
  $$RCI_{i}^{\left(k\right)}=\frac{DIS_{i}^{-\left(k\right)}}{DIS_{i}^{+\left(k\right)}+DIS_{i}^{-\left(k\right)}}$$
**Step-5**  To calculate the final revised closeness indices (FRCIs) by using the DMs weights as follows:
  $$FRCI_{i}=\sum_{k=1}^{e}Y^{\left(k\right)}\cdot RCI_{i}^{\left(k\right)}$$
  Rank the computed FRCIs values by descending order, the alternative has larger value is our most finest alternative.

## 5. Numerical Application of the Proposed Improved TOPSIS Method

In this section, an numerical application about selection of robot is firstly used to illustrate the designed MAGDM method. Then a comparison between the presented decision making technique and the existing decision making techniques using spherical fuzzy information are carried out to show the characteristic and advantage of the proposed technique.

### Example

A manufacturing unit needs a robot to play out a specific material-dealing task. The said model has been connected towards decision-making for choice of industrial robot carried out by the production unit of a famous manufacturing industry in Pakistan. After initial selection, five alternative robots, denoted as $S_{1}$, $S_{2}$, $S_{3}$, $S_{4}$, and $S_{5}$ have been selected for further scrutiny. A committee of three decision makers has been formed from academicians, manager of production unit and his team to locate the most suitable robot. The given set of criteria $\left\{f_{1},f_{2},f_{3},f_{4}\right\}$ have been considered. Where $f_{1}$ represents speediness, $f_{2}$ shows payload capacity, $f_{3}$ represents the programming flexibility and $f_{4}$ shows the Man-Machine interface. Where according to experts, attributes $f_{1}$ and $f_{3}$ are benefit type, $f_{2}$ and $f_{4}$ are cost type attributes. In this evaluation, the three experts were asked to use spherical fuzzy information and both, weights of DMs and attributes weights are unknown. The evaluation result of the experts is listed in Table 1, Table 2 and Table 3

**Step-1**  According to the experts, attribute $f_{1}$ and $f_{3}$ are benefits type, $f_{2}$ and $f_{4}$ are cost attributes. So, normalized matrix computed as given Formula (12), and results are shown in Table 4, Table 5 and Table 6
**Step-2**  GDIS matrix is computed as follows in Table 7:
**Step-2(b)**  GRID and GLID matrixes are computed as follows in Table 8 and Table 9:
**Step-2(c)**  DGDIS,DGRID and DGLID are computed as follows in Table 10, Table 11 and Table 12.
**Step-2(d)**  The closeness indices (CIs) are computed as follows:
$CI^{\left(1\right)}$$CI^{\left(2\right)}$$CI^{\left(3\right)}$0.7474850.7350900.717963
**Step-2(e)**  The Decision makers weights are computed as follows:
$Y^{\left(1\right)}$$Y^{\left(2\right)}$$Y^{\left(3\right)}$0.3400.3340.326
**Step-3(a)**  The revised group decision (RGDIS) matrix is computed as follows in Table 13.
**Step-3(b)**  SF entropy measure corresponding to each attribute is computed as follows:
$EA_{1}$$EA_{2}$$EA_{3}$$EA_{4}$0.335270.3949560.3592490.381971
**Step-3(c)**  The attribute weights are calculated as follows:
$\Phi A_{1}$$\Phi A_{2}$$\Phi A_{3}$$\Phi A_{4}$0.2630.2390.2530.245
**Step-4(a)**  The weighted normalized decision matrices are computed in Table 14, Table 15 and Table 16, as follows:
**Step-4(b)**  The $PIS^{\left(k\right)}$ and $NIS^{\left(k\right)}$ for each DMs are computed in Table 17 and Table 18, as follows:
**Step-4(c)**  Distance measure from positive ideal solution and negative ideal solution of each alternative are given in Table 19 and Table 20.
**Step-5**  The final revised closeness indices (FRCIs) by using the DMs weights are computed in Table 21, as follows:
  Hence, $S_{3}$ is the best alternative according to given attributes.

## 6. Comparison Analysis

In this section, a comparison of the characteristics of these proposed improved TOPSIS method and the designed MAGDM method is made to show the advantages of the designed technique. This comparison is carried out by comparing the characteristics of the different decision making technique presents in literature. In the method of [59], TOPSIS method for Pythagorean fuzzy information is presented. The Normalized DMs information are shown in Table 22, Table 23 and Table 24.

Decision Maker weights are computed as follows
$Y^{\left(1\right)}$$Y^{\left(2\right)}$$Y^{\left(3\right)}$0.3300.3540.316

Attributes weights are computed as follows
$\Phi A_{1}$$\Phi A_{2}$$\Phi A_{3}$$\Phi A_{4}$0.260.240.250.25

The final revised closeness indices (FRCIs) by using the DMs weights are computed in Table 25, as follows:

Hence, $S_{2}$ is the best alternative according to given attributes.

### Result and Discussion

The decision maker give the information in the form of Pythagorean fuzzy sets. In comparison section, we consider the neutral term equal to zero and used the proposed spherical improved TOPSIS technique to solve the information. As in the obtaining results, $S_{2}$ are the best alternative which is same as the given in the [59].

Here we gave some comparison of previously presented TOPSIS techniques and proposed improved TOPSIS technique is shown in Table 26.

Hence, as a consequence, the proposed methodology is more accurate, feasible, effective and generalized to solve MAGDM problems with completely unknown information among DMs as well as criteria.

## 7. Conclusions

SFS is an emerging and successful generalized notion that has been chosen as strategic tools to overcome the uncertainties as well as the vagueness data associated with MAGDM problems and therefore DMs feel more comfortable in their decision to use SF data rather than IFS, PyFS and PFS. In this paper, a novel improved TOPSIS-based decision-making method is established to deal the MAGDM problems under SF environment with completely unknown information about the DMs and criteria weights. GDM based novel SF entropy measure is proposed to establish the SF entropy weight model for computing the criteria weights under SF information. In order to eliminate the failure of collective information during the method, aggregation is performed in the last steps by using the computed weights of DMs to acquire the final alternative rank. Finally, numerical examples are illustrated to present the applicability and advantage of the introduced technique.

Because the spherical fuzzy numbers are very suitable for describing uncertain and fuzzy information, it is widely applied to real decision making, such as human resource management, online commodity recommendation, and so on. Meanwhile, the proposed technique can take relationships between attributes into account, it is more scientific to do decision making. In the future research, we will continue to focus on the extension and applications of more advanced decision making techniques to other realms.

## Figures and Tables

**Table 1:** DM${}_{1}$ information.

	$f_{1}$	$f_{2}$	$f_{3}$	$f_{4}$
$S_{1}$	$\left(0.84,0.34,0.40\right)$	$\left(0.43,0.39,0.78\right)$	$\left(0.67,0.50,0.30\right)$	$\left(0.31,0.21,0.71\right)$
$S_{2}$	$\left(0.60,0.11,0.53\right)$	$\left(0.23,0.35,0.59\right)$	$\left(0.72,0.31,0.41\right)$	$\left(0.11,0.25,0.82\right)$
$S_{3}$	$\left(0.79,0.19,0.39\right)$	$\left(0.11,0.21,0.91\right)$	$\left(0.71,0.40,0.13\right)$	$\left(0.34,0.25,0.51\right)$
$S_{4}$	$\left(0.63,0.51,0.13\right)$	$\left(0.49,0.33,0.42\right)$	$\left(0.61,0.43,0.45\right)$	$\left(0.49,0.37,0.59\right)$
$S_{5}$	$\left(0.57,0.36,0.29\right)$	$\left(0.50,0.15,0.60\right)$	$\left(0.70,0.32,0.40\right)$	$\left(0.33,0.44,0.65\right)$

**Table 2:** DM${}_{2}$ information.

	$f_{1}$	$f_{2}$	$f_{3}$	$f_{4}$
$S_{1}$	$\left(0.61,0.15,0.53\right)$	$\left(0.16,0.35,0.62\right)$	$\left(0.61,0.35,0.47\right)$	$\left(0.55,0.17,0.74\right)$
$S_{2}$	$\left(0.66,0.11,0.51\right)$	$\left(0.43,0.23,0.77\right)$	$\left(0.93,0.08,0.09\right)$	$\left(0.02,0.06,0.99\right)$
$S_{3}$	$\left(0.88,0.09,0.07\right)$	$\left(0.05,0.06,0.89\right)$	$\left(0.56,0.17,0.44\right)$	$\left(0.43,0.13,0.61\right)$
$S_{4}$	$\left(0.59,0.32,0.34\right)$	$\left(0.24,0.48,0.51\right)$	$\left(0.68,0.53,0.39\right)$	$\left(0.34,0.21,0.61\right)$
$S_{5}$	$\left(0.71,0.31,0.24\right)$	$\left(0.35,0.41,0.69\right)$	$\left(0.73,0.44,0.21\right)$	$\left(0.22,0.49,0.74\right)$

**Table 3:** DM${}_{2}$ information.

	$f_{1}$	$f_{2}$	$f_{3}$	$f_{4}$
$S_{1}$	$\left(0.85,0.25.0.15\right)$	$\left(0.14,0.23,0.88\right)$	$\left(0.78,0.38,0.18\right)$	$\left(0.29,0.39,0.83\right)$
$S_{2}$	$\left(0.94,0.04,0.07\right)$	$\left(0.39,0.19,0.61\right)$	$\left(0.63,0.18,0.35\right)$	$\left(0.48,0.49,0.56\right)$
$S_{3}$	$\left(0.73,0.13,0.46\right)$	$\left(0.19,0.39,0.88\right)$	$\left(0.87,0.35,0.18\right)$	$\left(0.41,0.13,0.81\right)$
$S_{4}$	$\left(0.82,0.12,0.43\right)$	$\left(0.55,0.21,0.63\right)$	$\left(0.53,0.33,0.47\right)$	$\left(0.46,0.23,0.51\right)$
$S_{5}$	$\left(0.61,0.33,0.29\right)$	$\left(0.28,0.41,0.63\right)$	$\left(0.74,0.34,0.14\right)$	$\left(0.37,0.32,0.65\right)$

**Table 4:** Normalized DM${}_{2}$ information.

	$f_{1}$	$f_{2}$	$f_{3}$	$f_{4}$
$S_{1}$	$\left(0.84,0.34,0.40\right)$	$\left(0.78,0.39,0.43\right)$	$\left(0.67,0.50,0.30\right)$	$\left(0.71,0.21,0.31\right)$
$S_{2}$	$\left(0.60,0.11,0.53\right)$	$\left(0.59,0.35,0.23\right)$	$\left(0.72,0.31,0.41\right)$	$\left(0.82,0.25,0.11\right)$
$S_{3}$	$\left(0.79,0.19,0.39\right)$	$\left(0.91,0.21,0.11\right)$	$\left(0.71,0.41,0.13\right)$	$\left(0.51,0.25,0.34\right)$
$S_{4}$	$\left(0.63,0.51,0.13\right)$	$\left(0.42,0.33,0.49\right)$	$\left(0.61,0.43,0.45\right)$	$\left(0.59,0.37,0.49\right)$
$S_{5}$	$\left(0.57,0.36,0.29\right)$	$\left(0.60,0.15,0.50\right)$	$\left(0.70,0.32,0.40\right)$	$\left(0.65,0.44,0.33\right)$

**Table 5:** Normalized DM${}_{2}$ information.

	$f_{1}$	$f_{2}$	$f_{3}$	$f_{4}$
$S_{1}$	$\left(0.61,0.15,0.53\right)$	$\left(0.62,0.35,0.16\right)$	$\left(0.61,0.35,0.47\right)$	$\left(0.74,0.17,0.55\right)$
$S_{2}$	$\left(0.66,0.11,0.51\right)$	$\left(0.77,0.23,0.43\right)$	$\left(0.93,0.08,0.09\right)$	$\left(0.99,0.06,0.02\right)$
$S_{3}$	$\left(0.88,0.09,0.07\right)$	$\left(0.89,0.06,0.05\right)$	$\left(0.56,0.17,0.44\right)$	$\left(0.61,0.13,0.43\right)$
$S_{4}$	$\left(0.59,0.32,0.34\right)$	$\left(0.51,0.48,0.24\right)$	$\left(0.68,0.53,0.39\right)$	$\left(0.61,0.21,0.34\right)$
$S_{5}$	$\left(0.71,0.31,0.24\right)$	$\left(0.69,0.41,0.35\right)$	$\left(0.73,0.44,0.21\right)$	$\left(0.74,0.49,0.22\right)$

**Table 6:** Normalized DM${}_{2}$ information.

	$f_{1}$	$f_{2}$	$f_{3}$	$f_{4}$
$S_{1}$	$\left(0.85,0.25,0.15\right)$	$\left(0.88,0.23,0.14\right)$	$\left(0.78,0.38,0.18\right)$	$\left(0.83,0.39,0.29\right)$
$S_{2}$	$\left(0.94,0.04,0.07\right)$	$\left(0.61,0.19,0.39\right)$	$\left(0.63,0.18,0.35\right)$	$\left(0.56,0.49,0.48\right)$
$S_{3}$	$\left(0.73,0.13,0.46\right)$	$\left(0.88,0.39,0.19\right)$	$\left(0.87,0.35,0.18\right)$	$\left(0.81,0.13,0.41\right)$
$S_{4}$	$\left(0.82,0.12,0.43\right)$	$\left(0.63,0.21,0.55\right)$	$\left(0.53,0.33,0.47\right)$	$\left(0.51,0.23,0.46\right)$
$S_{5}$	$\left(0.61,0.33,0.29\right)$	$\left(0.63,0.41,0.28\right)$	$\left(0.74,0.34,0.14\right)$	$\left(0.65,0.32,0.37\right)$

**Table 7:** GDIS Matrix

	$f_{1}$	$f_{2}$	$f_{3}$	$f_{4}$
$S_{1}$	$\left(0.792,0.233,0.316\right)$	$\left(0.788,0.315,0.212\right)$	$\left(0.697,0.405,0.293\right)$	$\left(0.766,0.240,0.367\right)$
$S_{2}$	$\left(0.807,0.078,0.278\right)$	$\left(0.670,0.248,0.342\right)$	$\left(0.812,0.164,0.234\right)$	$\left(0.913,0.194,0.101\right)$
$S_{3}$	$\left(0.811,0.130,0.232\right)$	$\left(0.894,0.170,0.101\right)$	$\left(0.751,0.290,0.217\right)$	$\left(0.676,0.161,0.391\right)$
$S_{4}$	$\left(0.703,0.269,0.266\right)$	$\left(0.532,0.321,0.401\right)$	$\left(0.613,0.422,0.435\right)$	$\left(0.573,0.261,0.424\right)$
$S_{5}$	$\left(0.636,0.332,0.272\right)$	$\left(0.642,0.293,0.365\right)$	$\left(0.723,0.363,0.227\right)$	$\left(0.683,0.410,0.299\right)$

**Table 8:** GRID matrix.

	$f_{1}$	$f_{2}$	$f_{3}$	$f_{4}$
$S_{1}$	$\left(0.85,0.25,0.15\right)$	$\left(0.88,0.23,0.14\right)$	$\left(0.78,0.38,0.18\right)$	$\left(0.71,0.21,0.31\right)$
$S_{2}$	$\left(0.94,0.04,0.08\right)$	$\left(0.77,0.23,0.43\right)$	$\left(0.93,0.08,0.09\right)$	$\left(0.99,0.06,0.02\right)$
$S_{3}$	$\left(0.88,0.09,0.07\right)$	$\left(0.89,0.06,0.05\right)$	$\left(0.87,0.35,0.18\right)$	$\left(0.81,0.13,0.41\right)$
$S_{4}$	$\left(0.82,0.12,0.43\right)$	$\left(0.63,0.21,0.55\right)$	$\left(0.68,0.53,0.39\right)$	$\left(0.61,0.21,0.34\right)$
$S_{5}$	$\left(0.71,0.31,0.24\right)$	$\left(0.60,0.15,0.50\right)$	$\left(0.74,0.34,0.14\right)$	$\left(0.74,0.49,0.22\right)$

**Table 9:** GLID Matrix.

	$f_{1}$	$f_{2}$	$f_{3}$	$f_{4}$
$S_{1}$	$\left(0.61,0.15,0.53\right)$	$\left(0.78,0.39,0.43\right)$	$\left(0.61,0.35,0.47\right)$	$\left(0.74,0.17,0.55\right)$
$S_{2}$	$\left(0.60,0.11,0.53\right)$	$\left(0.59,0.35,0.23\right)$	$\left(0.72,0.31,0.41\right)$	$\left(0.56,0.49,0.48\right)$
$S_{3}$	$\left(0.73,0.13,0.46\right)$	$\left(0.88,0.39,0.19\right)$	$\left(0.56,0.17,0.44\right)$	$\left(0.51,0.25,0.34\right)$
$S_{4}$	$\left(0.59,0.32,0.34\right)$	$\left(0.42,0.33,0.49\right)$	$\left(0.53,0.33,0.47\right)$	$\left(0.59,0.37,0.49\right)$
$S_{5}$	$\left(0.57,0.36,0.29\right)$	$\left(0.69,0.41,0.35\right)$	$\left(0.70,0.32,0.40\right)$	$\left(0.65,0.44,0.33\right)$

**Table 10:** DGDIS Matrix.

DM	$S_{1}$	$S_{2}$	$S_{3}$	$S_{4}$	$S_{5}$
$DM_{1}$	0.163292	0.322805	0.180650	0.192748	0.148069
$DM_{2}$	0.331040	0.294078	0.237022	0.197133	0.139253
$DM_{3}$	0.202054	0.500077	0.260579	0.229511	0.103838

**Table 11:** DGDIS Matrix.

DM	$S_{1}$	$S_{2}$	$S_{3}$	$S_{4}$	$S_{5}$
$DM_{1}$	0.251636	0.584725	0.371449	0.364557	0.198260
$DM_{2}$	0.492293	0.364128	0.395095	0.339937	0.170052
$DM_{3}$	0.151590	0.646511	0.249619	0.211065	0.237425

**Table 12:** DGLID Matrix.

DM	$S_{1}$	$S_{2}$	$S_{3}$	$S_{4}$	$S_{5}$
$DM_{1}$	0.330545	0.322439	0.238998	0.160350	0.159585
$DM_{2}$	0.195485	0.630518	0.268370	0.271129	0.198746
$DM_{3}$	0.463985	0.440680	0.443234	0.308255	0.146934

**Table 13:** DGLID Matrix.

	$f_{1}\;\;$	$f_{2}$	$f_{3}$	$f_{4}$
$S_{1}$	$\left(0.792,0.234,0.319\right)$	$\left(0.787,0.316,0.214\right)$	$\left(0.696,0.405,0.295\right)$	$\left(0.766,0.239,0.367\right)$
$S_{2}$	$\left(0.804,0.079,0.282\right)$	$\left(0.670,0.249,0.342\right)$	$\left(0.813,0.165,0.234\right)$	$\left(0.914,0.193,0.100\right)$
$S_{3}$	$\left(0.812,0.130,0.231\right)$	$\left(0.894,0.169,0.101\right)$	$\left(0.749,0.290,0.217\right)$	$\left(0.674,0.162,0.390\right)$
$S_{4}$	$\left(0.701,0.272,0.264\right)$	$\left(0.531,0.322,0.400\right)$	$\left(0.613,0.422,0.435\right)$	$\left(0.573,0.262,0.424\right)$
$S_{5}$	$\left(0.636,0.332,0.272\right)$	$\left(0.642,0.291,0.367\right)$	$\left(0.723,0.363,0.229\right)$	$\left(0.683,0.411,0.299\right)$

**Table 14:** Weighted Normalized DM${}_{1}$ information$\left(DM{\left(N\right)}_{ij}^{\left(1\right)}\right)$.

	$f_{1}\;\;$	$f_{2}$	$f_{3}$	$f_{4}$
$S_{1}$	$\left(0.275,0.752,0.785\right)$	$\left(0.200,0.798,0.817\right)$	$\left(0.139,0.839,0.737\right)$	$\left(0.157,0.682,0.750\right)$
$S_{2}$	$\left(0.110,0.559,0.846\right)$	$\left(0.097,0.778,0.703\right)$	$\left(0.168,0.743,0.798\right)$	$\left(0.239,0.712,0.582\right)$
$S_{3}$	$\left(0.226,0.646,0.780\right)$	$\left(0.343,0.688,0.590\right)$	$\left(0.162,0.798,0.596\right)$	$\left(0.071,0.712,0.767\right)$
$S_{4}$	$\left(0.124,0.837,0.584\right)$	$\left(0.045,0.767,0.843\right)$	$\left(0.111,0.807,0.817\right)$	$\left(0.099,0.783,0.839\right)$
$S_{5}$	$\left(0.098,0.764,0.722\right)$	$\left(0.101,0.635,0.847\right)$	$\left(0.156,0.749,0.793\right)$	$\left(0.125,0.817,0.762\right)$

**Table 15:** Weighted Normalized DM${}_{2}$ information $\left(DM{\left(N\right)}_{ij}^{\left(2\right)}\right)$.

	$f_{1}\;\;$	$f_{2}$	$f_{3}$	$f_{4}$
$S_{1}$	$\left(0.115,0.607,0.846\right)$	$\left(0.109,0.778,0.645\right)$	$\left(0.111,0.766,0.826\right)$	$\left(0.176,0.647,0.863\right)$
$S_{2}$	$\left(0.139,0.559,0.837\right)$	$\left(0.193,0.703,0.817\right)$	$\left(0.397,0.527,0.543\right)$	$\left(0.616,0.501,0.383\right)$
$S_{3}$	$\left(0.324,0.530,0.496\right)$	$\left(0.312,0.510,0.488\right)$	$\left(0.090,0.638,0.812\right)$	$\left(0.107,0.606,0.813\right)$
$S_{4}$	$\left(0.106,0.741,0.752\right)$	$\left(0.069,0.839,0.711\right)$	$\left(0.145,0.851,0.788\right)$	$\left(0.107,0.682,0.767\right)$
$S_{5}$	$\left(0.168,0.734,0.687\right)$	$\left(0.143,0.808,0.778\right)$	$\left(0.175,0.812,0.673\right)$	$\left(0.176,0.839,0.690\right)$

**Table 16:** Weighted Normalized DM${}_{3}$ information $\left(DM{\left(N\right)}_{ij}^{\left(3\right)}\right)$.

	$f_{1}\;\;$	$f_{2}$	$f_{3}$	$f_{4}$
$S_{1}$	$\left(0.286,0.694,0.607\right)$	$\left(0.299,0.703,0.625\right)$	$\left(0.211,0.782,0.648\right)$	$\left(0.248,0.793,0.738\right)$
$S_{2}$	$\left(0.432,0.428,0.514\right)$	$\left(0.105,0.672,0.798\right)$	$\left(0.120,0.648,0.766\right)$	$\left(0.088,0.839,0.835\right)$
$S_{3}$	$\left(0.181,0.584,0.815\right)$	$\left(0.299,0.798,0.672\right)$	$\left(0.300,0.766,0.648\right)$	$\left(0.230,0.606,0.803\right)$
$S_{4}$	$\left(0.254,0.572,0.800\right)$	$\left(0.113,0.688,0.866\right)$	$\left(0.080,0.755,0.826\right)$	$\left(0.071,0.697,0.826\right)$
$S_{5}$	$\left(0.115,0.747,0.722\right)$	$\left(0.113,0.808,0.737\right)$	$\left(0.181,0.761,0.608\right)$	$\left(0.125,0.756,0.783\right)$

**Table 17:** Positive ideal solution for each DMs.

	$f_{1}$	$f_{2}$	$f_{3}$	$f_{4}$
$PIS^{\left(1\right)}$	$\left(0.226,0.646,0.780\right)$	$\left(0.343,0.688,0.590\right)$	$\left(0.162,0.798,0.596\right)$	$\left(0.239,0.712,0.582\right)$
$PIS^{\left(2\right)}$	$\left(0.324,0.530,0.496\right)$	$\left(0.312,0.510,0.488\right)$	$\left(0.397,0.527,0.543\right)$	$\left(0.616,0.501,0.383\right)$
$PIS^{\left(3\right)}$	$\left(0.432,0.428,0.514\right)$	$\left(0.299,0.703,0.625\right)$	$\left(0.300,0.766,0.648\right)$	$\left(0.230,0.606,0.803\right)$

**Table 18:** Negative ideal solution for each DMs.

	$f_{1}$	$f_{2}$	$f_{3}$	$f_{4}$
$NIS^{\left(1\right)}$	$\left(0.098,0.764,0.722\right)$	$\left(0.045,0.767,0.843\right)$	$\left(0.111,0.807,0.817\right)$	$\left(0.099,0.783,0.839\right)$
$NIS^{\left(2\right)}$	$\left(0.106,0.741,0.752\right)$	$\left(0.069,0.839,0.711\right)$	$\left(0.145,0.851,0.788\right)$	$\left(0.176,0.839,0.690\right)$
$NIS^{\left(3\right)}$	$\left(0.115,0.747,0.722\right)$	$\left(0.113,0.688,0.866\right)$	$\left(0.080,0.755,0.826\right)$	$\left(0.088,0.839,0.835\right)$

**Table 19:** Distance from positive ideal solution.

	$S_{1}$	$S_{2}$	$S_{3}$	$S_{4}$	$S_{5}$
$DIS_{i}^{{+}^{\left(1\right)}}$	0.17493	0.14188	0.08923	0.26340	0.20944
$DIS_{i}^{{+}^{\left(2\right)}}$	0.38196	0.23932	0.26906	0.37982	0.35833
$DIS_{i}^{{+}^{\left(3\right)}}$	0.15668	0.17445	0.17409	0.22637	0.20918

**Table 20:** Distance from negative ideal solution.

	$S_{1}$	$S_{2}$	$S_{3}$	$S_{4}$	$S_{5}$
$DIS_{i}^{{-}^{\left(1\right)}}$	0.09975	0.19918	0.19788	0.07792	0.08702
$DIS_{i}^{{-}^{\left(2\right)}}$	0.17628	0.33558	0.29697	0.09260	0.08275
$DIS_{i}^{{-}^{\left(3\right)}}$	0.18343	0.19163	0.21808	0.12273	0.15634

**Table 21:** Final revised closeness indices.

Alternatives	$S_{1}$	$S_{2}$	$S_{3}$	$S_{4}$	$S_{5}$
FRCIs	0.40476	0.56417	0.59085	0.25769	0.30189

**Table 22:** Normalized DM${}_{1}$ information.

	$f_{1}\;\;$	$f_{1}\;\;$	$f_{1}\;\;$	$f_{1}\;\;$
$S_{1}$	$\left(0.4,0.8\right)$	$\left(0.8,0.6\right)$	$\left(0.6,0.7\right)$	$\left(0.3,0.8\right)$
$S_{2}$	$\left(0.7,0.5\right)$	$\left(0.9,0.2\right)$	$\left(0.8,0.5\right)$	$\left(0.3,0.6\right)$
$S_{3}$	$\left(0.3,0.4\right)$	$\left(0.3,0.7\right)$	$\left(0.7,0.4\right)$	$\left(0.6,0.4\right)$
$S_{4}$	$\left(0.6,0.6\right)$	$\left(0.7,0.5\right)$	$\left(0.7,0.2\right)$	$\left(0.4,0.6\right)$
$S_{5}$	$\left(0.5,0.7\right)$	$\left(0.6,0.4\right)$	$\left(0.9,0.3\right)$	$\left(0.6,0.7\right)$

**Table 23:** Normalized DM${}_{2}$ information.

	$f_{1}\;\;$	$f_{1}\;\;$	$f_{1}\;\;$	$f_{1}\;\;$
$S_{1}$	$\left(0.3,0.9\right)$	$\left(0.7,0.6\right)$	$\left(0.5,0.8\right)$	$\left(0.3,0.6\right)$
$S_{2}$	$\left(0.7,0.4\right)$	$\left(0.9,0.2\right)$	$\left(0.8,0.1\right)$	$\left(0.3,0.5\right)$
$S_{3}$	$\left(0.3,0.6\right)$	$\left(0.7,0.7\right)$	$\left(0.7,0.6\right)$	$\left(0.4,0.4\right)$
$S_{4}$	$\left(0.4,0.8\right)$	$\left(0.7,0.5\right)$	$\left(0.6,0.2\right)$	$\left(0.4,0.7\right)$
$S_{5}$	$\left(0.2,0.7\right)$	$\left(0.8,0.2\right)$	$\left(0.8,0.4\right)$	$\left(0.6,0.6\right)$

**Table 24:** Normalized DM${}_{3}$ information.

	$f_{1}\;\;$	$f_{1}\;\;$	$f_{1}\;\;$	$f_{1}\;\;$
$S_{1}$	$\left(0.6,0.8\right)$	$\left(0.7,0.6\right)$	$\left(0.5,0.8\right)$	$\left(0.5,0.5\right)$
$S_{2}$	$\left(0.6,0.5\right)$	$\left(0.9,0.2\right)$	$\left(0.8,0.1\right)$	$\left(0.3,0.5\right)$
$S_{3}$	$\left(0.4,0.7\right)$	$\left(0.7,0.5\right)$	$\left(0.6,0.1\right)$	$\left(0.2,0.9\right)$
$S_{4}$	$\left(0.2,0.9\right)$	$\left(0.5,0.6\right)$	$\left(0.6,0.2\right)$	$\left(0.1,0.6\right)$
$S_{5}$	$\left(0.1,0.6\right)$	$\left(0.8,0.2\right)$	$\left(0.9,0.2\right)$	$\left(0.6,0.5\right)$

**Table 25:** Final revised closeness indices.

Alternatives	$S_{1}$	$S_{2}$	$S_{3}$	$S_{4}$
FRCIs	0.205	0.842	0.457	0.538

**Table 26:** Comparison Analysis.

Scholars	Uncertainty Approach			Modeling Approach		$\begin{array}{c} \mathrm{Unknown}\;\mathrm{Weights}\\ \mathrm{Information} \end{array}$	
	**FSs**	**PyFs**	**SFSs**	$\begin{array}{c} \mathrm{Group}\\ \mathrm{Decision}\\ \mathrm{Making} \end{array}$	$\begin{array}{c} \mathrm{TOPSIS}\\ \mathrm{Method} \end{array}$	$\begin{array}{c} \mathrm{Decision}\\ \mathrm{Maker} \end{array}$	**Attributes**
Beg and Rashid [39]	yes	no	no	no	yes	no	no
Zhang and Xu [49]	yes	yes	no	no	yes	no	no
Yue [56]	yes	no	no	yes	no	yes	no
Proposed Technique	yes	yes	yes	yes	yes	yes	yes
